# Dermatological manifestations and skin care practices associated with self-efficacy among adults with type 2 diabetes mellitus in Qassim region, Saudi Arabia: a multicenter study

**DOI:** 10.3389/fpubh.2026.1846781

**Published:** 2026-06-29

**Authors:** Mohamed Goda Elbqry, Fatma Mohamed Elmansy, Shereen Ahmed A. Qalawa, Moodhi Mohammed Alolayan, Aljoud Abdulrahman Alshitwy, Samia Eaid Elgazzar, Mona Abdullah Mohamed Ali, Enas Ebrahiem Elsayed, Manal Th. Soliman

**Affiliations:** 1 Department of Medical-Surgical Nursing, College of Nursing, Qassim University, Buraydah, Saudi Arabia; 2 Department of Nursing, Medical City, Qassim University, Buraydah, Saudi Arabia; 3 Department of Nursing, Al Midhnab General Hospital, Buraydah, Saudi Arabia; 4 Department of Medical-Surgical Nursing, Mansoura University, Mansoura, Egypt

**Keywords:** dermatological manifestations, glycemic control, multicenter study, Qassim region, self-efficacy, skin care practices, T2DM

## Abstract

**Background:**

Type 2 Diabetes Mellitus (T2DM) is a major global public health concern characterized by chronic hyperglycemia and associated complications, including dermatological manifestations. Skin disorders are common among patients with diabetes and are often linked to poor glycemic control and inadequate self-care practices. Self-efficacy is a key determinant of effective diabetes self-management and may influence the occurrence and severity of dermatological conditions.

**Aim:**

This study aimed to examine the association between dermatological manifestations, skin care practices, and self-efficacy among adults with T2DM in the Qassim region, Saudi Arabia.

**Methods:**

A cross-sectional study was conducted among adult patients (30–70 years) with T2DM in Saudi Arabia using a convenience sampling approach. Data were collected through an online structured self-administered questionnaire distributed via digital platforms. The questionnaire included three components: (1) sociodemographic and clinical characteristics, including glycemic indicators; (2) skin care practices; and (3) self-efficacy in diabetes self-management assessed using a 7-item scale. Data were analyzed using descriptive statistics and inferential tests to assess associations between variables.

**Results:**

Participants demonstrated suboptimal glycemic control, with a considerable proportion reporting HbA1c levels between 7.6–8.5% and ≥9.6%. The median (IQR) fasting blood glucose was 111.0 (99.0–125.0) mg/dL, and random blood glucose was 191.0 (163.0–212.0) mg/dL. Self-efficacy levels were significantly associated with most sociodemographic characteristics (*p* < 0.001). Significant associations were observed between self-efficacy and multiple dermatological manifestations, including ulcers, nail abnormalities, itching, tenderness, redness, paronychia, hyperpigmentation, rashes, and purulent lesions (*p* < 0.001). A significant association was also found with swelling (*p* = 0.040), while no significant association was identified with vascular impairment (*p* = 0.249).

**Conclusion:**

Self-efficacy is significantly associated with dermatological manifestations among patients with T2DM. Strengthening patient education and behavioral interventions targeting glycemic control and skin care practices may enhance self-efficacy and reduce diabetes-related skin complications.

## 1 Introduction

Diabetes Mellitus is a major global public health challenge characterized by persistent hyperglycemia resulting from defects in insulin secretion, insulin action, or both. Over time, uncontrolled diabetes leads to serious complications affecting multiple organ systems, including the cardiovascular system, kidneys, eyes, nerves, and skin. The global burden of diabetes has increased markedly in recent decades, with projections indicating a continued rise, particularly in low- and middle-income countries (1).

Among its complications, dermatological manifestations are common yet frequently under recognized in routine clinical practice. These manifestations may represent early indicators of underlying metabolic dysregulation, including chronic hyperglycemia, insulin resistance, and microvascular impairment, or may reflect the severity and duration of the disease (2). Skin changes such as infections, xerosis, pruritus, and pigmentary alterations are often the first clinical signs observed in patients with T2DM and may precede the diagnosis of diabetes or signal poor glycemic control. Moreover, these conditions can significantly impact patients' quality of life and increase the risk of more serious complications, such as ulceration and infection (3).

T2DM, the most prevalent form of diabetes, has reached epidemic proportions worldwide. Dermatological manifestations have been reported in up to 30% of patients and may occur at disease onset or during disease progression (1). These manifestations include a wide spectrum of conditions, such as diabetic dermopathy, necrobiosis lipoidica, acanthosis nigricans, infections, microvascular and macrovascular skin changes, and adverse reactions to antidiabetic therapies (4). In Saudi Arabia, diabetes represents a significant public health concern, with approximately 7 million individuals affected and an additional 3 million at risk due to prediabetes (1). This substantial burden highlights the urgent need for improved prevention and management strategies targeting diabetes-related complications, including dermatological manifestations (5).

Evidence suggests that dermatological manifestations are more prevalent and severe among patients with T2DM, particularly in those with prolonged disease duration and poor glycemic control. Complications such as diabetic foot ulcers, primarily driven by peripheral neuropathy and vascular impairment, significantly increase the risk of infection, amputation, and reduced quality of life (6). Despite their clinical significance, dermatological complications are often inadequately addressed in routine diabetes care (7).

From a behavioral perspective, effective diabetes management relies heavily on patients' engagement in self-care practices. Self-efficacy, as described in Self-Efficacy Theory, refers to an individual's belief in their ability to perform behaviors necessary to achieve desired outcomes. In diabetes care, self-efficacy plays a crucial role in influencing adherence to treatment, glycemic control, foot care, and preventive behaviors (8). Higher levels of self-efficacy have been consistently associated with improved self-management and better clinical outcomes (3).

Although previous studies have extensively examined dermatological manifestations and glycemic control in patients with diabetes, limited research has investigated the relationship between dermatological complications and behavioral determinants such as self-efficacy, particularly within the Saudi context (4). Moreover, most existing studies focus primarily on clinical outcomes without integrating psychosocial frameworks that explain patient behavior. Grounded in Bandura's Self-Efficacy Theory, this study addresses this gap by examining how patients' confidence in their self-care abilities relates to the occurrence and management of dermatological manifestations (6). Therefore, this study aimed to examine the association between dermatological manifestations, skin care practices, and self-efficacy among adults with T2DM in the Qassim region, Saudi Arabia.

Research questions:

1. What are the common dermatological manifestations among adults with T2DM?
2. What are the skin care practices level among adults with T2DM?
3. Is there an association between dermatological manifestations and self-efficacy among adults with T2DM?
4. Is there an association between sociodemographic characteristics and self-efficacy among adults with T2DM?
5. What are the significant predictors of self-efficacy among adults with T2DM?

## 2 Method

### 2.1 Study design

A descriptive cross-sectional study was conducted in this study.

### 2.2 Study participants and setting

The study population comprised adults diagnosed with T2DM from different cities within the Qassim region, Saudi Arabia. Participants were recruited using a convenience sampling technique through online platforms, including community-based forums, and community networks to maximize outreach across different regions of Saudi Arabia to maximize outreach and participation.

Eligibility criteria included adults aged 30–70 years, of both sexes, with a self-reported medical diagnosis of T2DM, the ability to read and understand the Arabic questionnaire, access to an electronic device with internet connectivity, and willingness to participate voluntarily in the study. To ensure eligibility, the first section of the online questionnaire included screening questions related to age, diabetes diagnosis, and informed consent. Only participants who met the inclusion criteria were able to proceed with the questionnaire. Individuals who declined participation, were younger than 30 years or older than 70 years, had no confirmed diagnosis of T2DM, or demonstrated incomplete, inconsistent, or illogical response patterns were excluded from the analysis. Since the questionnaire was self-administered electronically, individuals with severe cognitive impairment or inability to independently complete the survey were considered ineligible for participation.

The required sample size was calculated using the standard formula for cross-sectional studies *n* = *Z*^2^*p*(1−*p*)/*d*^2^, assuming a 95% confidence level (Z = 1.96), an expected proportion of 50% (*p* = 0.50), and a margin of error of 5% (d = 0.05), yielding a minimum sample size of 384 participants. To account for the region scope and potential variability across it, a design effect of 2 was applied, resulting in an adjusted minimum sample size of 768 participants. A total of 2,011 participants were included in the final analysis, exceeding the minimum requirement and enhancing the precision and generalizability of the findings. The response rate was 80.4%, calculated based on 2,011 completed responses out of 2,500 eligible participants. *Post hoc* power analysis confirmed that the achieved sample size provided adequate statistical power (>80%) to detect significant associations at a 5% significance level.

### 2.3 Data collection tools

Data were collected using a structured, self-administered online questionnaire developed via Google Forms and distributed through websites and community-based digital platforms (e.g., forums and social media) to reach participants across the Qassim region, Saudi Arabia. The questionnaire was adapted from previously validated instruments (3–9). It consisted of three main sections.

#### 2.3.1 Section I: dermatological clinical manifestations sheet

It was used to assess participants' sociodemographic and clinical characteristics, including age, gender, occupation, duration of diabetes, and educational level. It also assessed dermatological manifestations, such as itching, redness, tenderness, hyperpigmentation, rashes, and nail changes. These manifestations were evaluated through self-reported responses rather than direct clinical examination; therefore, the reported conditions reflected participants' perceived symptoms and experiences. In addition, glycemic control indicators, including glycated hemoglobin (HbA1c), fasting blood glucose, and random blood glucose levels, were assessed. Participants were instructed to report their most recent laboratory investigation results, by referring to their medical records, laboratory reports, or electronic health applications whenever available. Dermatological manifestations were measured using a 3-point Likert scale ranging from 1 (never) to 3 (always), with higher scores indicating greater frequency or severity of symptoms.

#### 2.3.2 Section II: Skin care practices scale

Ten-Items scale, used to evaluate the studied patients' skin care practices using an adapted tool. This section included items related to foot care and hygiene behaviors, such as regular foot examination, appropriate nail care, use of socks, and frequency of foot washing. Responses were measured using a 5-point Likert scale (1 = never, 2 = rarely, 3 = sometimes, 4 = often, 5 = always), with higher scores indicating more adequate skin care practices (3–6).

#### 2.3.3 Section III: self-efficacy in diabetes self-management scale

7-Items scale utilized to measure the studied patients' self-efficacy adapted from previous related study, covering domains such as diet, physical activity, adherence to follow-up, and lifestyle control. Each item was rated on a 3-point Likert scale (= low confidence, 2 = moderate confidence, 3 = high confidence). The total self-efficacy score was calculated by summing item responses and converting them into percentage values. Based on these scores, self-efficacy levels were categorized as low (< 50%), moderate (50% to < 75%), and high (≥75%), with higher scores indicating greater self-efficacy in diabetes self-management (9).

#### 2.3.4 Pilot study

It was conducted on approximately 10% of the target sample to assess the clarity, feasibility, and applicability of the data collection instrument. Feedback from participants was used to refine the wording, structure, and sequencing of the questionnaire items. Participants included in the pilot study were excluded from the final analysis to avoid potential bias and ensure data accuracy.

#### 2.3.5 Validity of the instrument

It was established through expert evaluation by a panel of specialists in medical-surgical nursing, dermatology, and public health. The experts assessed the relevance, clarity, comprehensiveness, and cultural appropriateness of each item. Based on their feedback, necessary modifications were made to enhance the quality of the tool. The overall Content Validity Index (CVI) for the instrument was 0.91, indicating excellent content validity.

#### 2.3.6 Reliability of the instrument

It was assessed using Cronbach's alpha coefficient to evaluate internal consistency. The results demonstrated good reliability, with a Cronbach's alpha of 0.82 for the skin care practices scale and 0.88 for the self-efficacy in diabetes self-management scale. These values indicate a high level of internal consistency and reliability of the study instrument.

### 2.4 Data collection procedure

Data collection was conducted between May and October 2025 following the acquisition of informed consent from all participants. Ethical approval was obtained from the Research Ethics Committee of the Deanship of Scientific Research, Qassim University, Saudi Arabia (Approval No. 25-36-12; dated 08 May 2025), and all study procedures adhered to the principles of the Declaration of Helsinki. Prior to the main data collection, a pilot study was conducted on the target sample to evaluate the clarity, feasibility, and applicability of the instrument. Based on the pilot findings and expert feedback, minor modifications were made to improve the wording and structure of the questionnaire. The reliability and validity of the adapted instrument were confirmed prior to its final use.

The finalized questionnaire was distributed electronically via Google Forms using a convenience sampling approach. Participants were recruited through commonly used social media and community-based digital platforms in Saudi Arabia, including public online forums related to diabetes and community health. In addition, and through community networks to maximize outreach across different regions of Saudi Arabia. Potential participants were approached through invitation messages containing a brief explanation of the study purpose, eligibility criteria, voluntary nature of participation, and a secure survey link. Participants were also encouraged to share the survey with other eligible individuals diagnosed with T2DM.

To ensure eligibility, the first section of the questionnaire included screening questions related to age, diagnosis of T2DM, and willingness to participate. Only participants who met the inclusion criteria were able to proceed with the survey. Since the questionnaire was self-administered electronically, individuals with severe cognitive impairment were considered unlikely to independently complete the survey. Furthermore, participants were required to read and understand the study information and provide informed consent before accessing the questionnaire items. Responses showing incomplete, inconsistent, or illogical response patterns were carefully reviewed and excluded to minimize invalid responses and improve data quality.

To enhance participants' understanding of dermatological manifestations, simplified descriptions and commonly used non-medical terminology were provided alongside selected medical terms within the questionnaire. Brief explanatory of common skin symptoms and conditions were included to facilitate accurate self-identification and improve response accuracy. The clarity and comprehensibility of these items were further evaluated during the pilot study prior to the main data collection. An introductory cover note accompanied the questionnaire and outlined the study objectives, emphasized voluntary participation, and informed participants of their right to withdraw at any stage before submitting the questionnaire without penalty. The estimated time required to complete the questionnaire was approximately 3–5 min. To facilitate communication and address participants' inquiries, dedicated chatting groups and direct communication channels with the research team were made available throughout the data collection period. Completion of the questionnaire was considered as implied informed consent, and it was monitored for completeness. Participants were assured that all information would remain confidential and used solely for research purposes.

### 2.5 Statistical analysis

Data were coded and analyzed using IBM SPSS Statistics version 25.0 (IBM Corp., Armonk, NY, USA). Missing data were minimal (< 5%); therefore, incomplete responses were excluded, and no imputation was performed. Descriptive statistics were used to summarize the data, with categorical variables presented as frequencies and percentages, and continuous variables as mean ± standard deviation (SD) or median and interquartile range (IQR), as appropriate. Normality was assessed using the Kolmogorov–Smirnov test. Inferential analyses included the Chi-square test to examine associations between categorical variables. Multiple linear regression analysis was performed to identify independent predictors of self-efficacy, with self-efficacy score as the dependent variable and age, gender, educational level, duration of diabetes, dermatological manifestations score, and skin care practices score as independent variables. Regression coefficients (B), standardized beta coefficients (β), standard errors (SE), 95% confidence intervals (CI), and model fit indices (R^2^ and adjusted R^2^) were reported. All tests were two-tailed, and a *p*-value < 0.05 was considered statistically significant.

## 3 Results

Table 1 displays that the study included a total of 2,011 participants, with a slightly higher proportion of males (52.8%) compared to females (47.2%). The majority of participants were of Saudi nationality (74.3%), while 25.7% were non-Saudi. Most participants were employed (75.9%), whereas 24.1% were not working. Regarding marital status, 45.9% of participants were married. In terms of educational level, 21.9% had attained university education.

**Table 1:** Distribution of the study participants according to their sociodemographic characteristics (*n* = 2,011).

Socio-demographic data	*n* (%)
Age (years)	
30–35	415 (20.6)
35–45	613 (30.5)
45–55	502 (25.0)
55–65	240 (11.9)
More than 65	241 (12.0)
Gender	
Male	1,061 (52.8)
Female	950 (47.2)
Education	
Read and write	323 (16.1)
Primary	334 (16.6)
Secondary	360 (17.9)
Tertiary	282 (14.0)
University	440 (21.9)
Postgraduate	272 (13.5)
Occupation	
Not working	484 (24.1)
Working	1,527 (75.9)
Are you suffered any skin problems	
Yes	1,201 (59.7)
No	810 (40.3)

IQR, Inter quartile range; SD, Standard deviation.

Table 2 shows that 39.2% of participants had a duration of diabetes mellitus ranging from 7 to 10 years. Regarding treatment modality, the majority were receiving insulin therapy (63.6%), while 36.4% were treated with oral hypoglycemic agents. Concerning glycemic control, 22.9% of participants had HbA1c levels between 7.6% and 8.5%, followed by 20.7% with HbA1c levels ≥9.6%. The median (IQR) fasting blood glucose was 111.0 (99.0–125.0) mg/dL, and the median (IQR) random blood glucose was 191.0 (163.0–212.0) mg/dL.

**Table 2:** Distribution of the study participants according to diabetes-related clinical and glycemic characteristics (*n* = 2,011).

Diabetes mellitus data (*n* = 2,011)	*n* (%)
Duration of DM (years)	
5–7	604(30.0)
7–10	789 (39.2)
>10	618 (30.7)
Type of treatment	
Oral hypoglycemic drug	732 (36.4)
Insulin	1,279 (63.6)
Average of your HbA1 (%)	
≤ 6.5	335 (16.7)
6.6–7.5	403 (20.0)
7.6–8.5	461 (22.9)
8.6–9.5	396 (19.7)
≥9.6	416 (20.7)
Fasting blood sugar	
Min–max	86.4–200.0
Mean ± SD	111.8 ± 16.2
Median (IQR)	111.0 (99.0 −125.0)
Random blood sugar	
Min–max	85.0–240.0
Mean ± SD	189.4 ± 30.02
Median (IQR)	191.0 (163.0–212.0)

IQR, Inter quartile range, SD; Standard deviation; HbA1c, glycated hemoglobin.

Table 3 presents the distribution of dermatological manifestations among the study participants with Type 2 Diabetes Mellitus. The findings indicate that dermatological symptoms were common and varied in frequency across participants. Tenderness was the most frequently reported manifestation in the “Always” category (39.9%), followed by vascular impairment (38.2%), skin hyperpigmentation (38.0%), redness (36.6%), and itching (36.2%). Paronychia (39.0%) and rashes (38.0%) were the most commonly reported conditions in the “Sometimes” category. Regarding the “Never” category, ulceration showed the highest proportion (37.9%), followed by swelling (35.0%). Overall, the findings demonstrate a substantial burden of dermatological manifestations among patients with Type 2 Diabetes Mellitus, highlighting the importance of regular skin assessment and preventive skin care practices in this population.

**Table 3:** Distribution of the study participants according to dermatological manifestations (*n* = 2,011).

Skin disorder manifestations	Always		Sometimes		Never	
	*n*	%	*n*	%	*n*	%
Ulcer	667	33.2	581	28.9	763	37.9
Swelling	652	32.4	655	32.6	704	35.0
Yellow nails	644	32.0	704	35.0	663	33.0
Slow nail growth	702	34.9	660	32.8	649	32.3
Itching	727	36.2	722	35.9	562	27.9
Insomnia	675	33.5	667	33.2	669	33.3
Tenderness	802	39.9	566	28.1	643	32.0
Redness	737	36.6	648	32.2	626	31.2
Paronychia	623	31.0	784	39.0	604	30.0
Vascular impairment	768	38.2	690	34.3	553	27.5
Skin hyperpigmentation	765	38.0	597	29.7	649	32.3
Pus	701	34.8	681	33.9	629	31.3
Rashes	692	34.4	765	38.0	554	27.6

Table 4 presents that a high proportion of participants reported regularly examining their feet, with (69.3%) indicating they always perform this practice. Similarly, (57.0%) reported always washing their feet daily, and (60.7%) reported bathing regularly. More than half of the participants (54.9%) indicated appropriate use of nail scissors for foot care. However, some practices were less consistently followed. Only (19.1%) reported always wearing socks, while a considerable proportion (41.8%) reported rarely doing so. Cleaning the skin with a disinfectant before injection showed varied responses, with (20.5%) always performing this practice and (36.3%) never doing so. Additionally, (55.4%) of participants reported regularly examining their skin for abnormalities. Regarding daily hygiene behaviors, (34.5%) reported always washing their feet as part of their routine, and (46.3%) indicated that they consistently dried their feet thoroughly after washing. Furthermore, (39.0%) of participants reported always checking their footwear before wearing it.

**Table 4:** Distribution of the study participants according to skin care practices (*n* = 2,011).

Items	Always (5) *n* (%)	Often (4) *n* (%)	Sometimes (3) *n* (%)	Rarely (2) *n* (%)	Never (1) *n* (%)
1) Do you examine your feet regularly?	1,393 (69.3)	0 (0.0%)	0 (0.0%)	0 (0.0%)	618 (30.7)
2) Do you use nail scissors appropriately for foot care?	1,104 (54.9)	0 (0.0%)	479 (23.8)	0 (0.0%)	428 (21.3)
3) Do you wear socks regularly to protect your feet?	384 (19.1)	270 (13.4)	320 (15.9)	840 (41.8)	197 (9.8)
4) How often do you wash your feet daily?	1,147 (57.0)	0 (0.0%)	0 (0.0%)	0 (0.0%)	864 (43.0)
5) Do you clean the skin with a disinfectant (e.g., alcohol swab) before injection?	412 (20.5)	460 (22.9)	410 (20.4)	0 (0.0%)	729 (36.3)
6) Do you bathe regularly (at least once per week)?	1,220 (60.7%)	240 (11.9%)	182 (9.1%)	260 (12.9%)	109 (5.4%)
7) Do you examine your skin generally regularly for any abnormalities?	1,115 (55.4%)	229 (11.4%)	225 (11.2%)	200 (9.9%)	242 (12.0%)
8) Do you wash your feet regularly as part of your daily hygiene routine?	694 (34.5%)	499 (24.8%)	426 (21.2%)	166 (8.3%)	226 (11.2%)
9) Do you dry your feet thoroughly, especially between the toes, after washing?	931 (46.3%)	280 (13.9%)	183 (9.1%)	331 (16.5%)	286 (14.2%)
10) Do you check your footwear before wearing it?	785 (39.0%)	616 (30.6%)	63 (3.1%)	286 (14.2%)	261 (13.0%)

Table 5 shows the association between sociodemographic characteristics and self-efficacy levels among patients with type 2 diabetes. A statistically significant association was observed between age and self-efficacy levels (χ^2^ = 94.713, *p* < 0.001). Higher proportions of participants aged 30–35 years demonstrated high self-efficacy [50.4% (*n* = 2090)], whereas lower levels were more evident among older age groups. Gender was also significantly associated with self-efficacy (χ^2^ = 26.054, *p* < 0.001), with females showing a higher proportion of high self-efficacy [50.3% (*n* = 478)] compared to males [42.5% (*n* = 451)]. Educational level demonstrated a strong significant association with self-efficacy (χ^2^ = 175.832, *p* < 0.001). Participants with higher education levels, particularly university education, showed higher self-efficacy [49.1% (*n* = 216)], whereas those with intermediate education had comparatively lower levels [29.4% (*n* = 106)]. In contrast, no statistically significant association was found between occupation and self-efficacy levels (χ^2^ = 1.001, *p* = 0.606), although slightly higher proportions of high self-efficacy were observed among working participants [46.4% (*n* = 709)] compared to those not working [45.5% (*n* = 220)]. Additionally, the duration of diabetes mellitus was significantly associated with self-efficacy (χ^2^ = 37.484, *p* < 0.001). Participants with longer disease duration (>10 years) demonstrated higher self-efficacy levels [49.2% (*n* = 304)] compared to those with shorter durations.

**Table 5:** Association between self-efficacy levels and sociodemographic characteristics among patients with type 2 diabetes mellitus (*n* = 2,011).

Socio-demographic data	Level of self-efficacy care scale						χ^2^	*p*
	Low (< 50%) (*n* = 465)		Moderate (50%−75%) (*n* = 617)		High (>75%) (*n* = 929)			
	*n*	%	*n*	%	*n*	%		
Age (years)								
30–35	62	14.9	144	34.7	209	50.4	94.713^*^	< 0.001^*^
35–45	130	21.2	131	21.4	352	57.4		
45–55	121	24.1	183	36.5	198	39.4		
55–65	78	32.5	89	37.1	73	30.4		
More than 65	74	30.7	70	29.0	97	40.2		
Gender								
Male	232	21.9	378	35.6	451	42.5	26.054^*^	< 0.001^*^
Female	233	24.5	239	25.2	478	50.3		
Education								
Read and write	19	5.9	130	40.2	174	53.9	175.832^*^	< 0.001^*^
Primary	66	19.8	59	17.7	209	62.6		
Intermediate	141	39.2	113	31.4	106	29.4		
Secondary	64	22.7	95	33.7	123	43.6		
University	87	19.8	137	31.1	216	49.1		
Postgraduate	88	32.4	83	30.5	101	37.1		
Occupation								
Not working	107	22.1	157	32.4	220	45.5	1.001	0.606
Working	358	23.4	460	30.1	709	46.4		
Duration of DM (years)								
5–7	182	30.1	146	24.2	276	45.7	37.484^*^	< 0.001^*^
7–10	153	19.4	287	36.4	349	44.2		
>10	130	21.0	184	29.8	304	49.2		

**χ^2^**, Chi square test; ***p***: Statistically significant at *p* ≤ 0.05. ^*^Means statistically significant.

Table 6 demonstrates the association between self-efficacy levels and dermatological manifestations among patients with Type 2 Diabetes Mellitus. Significant associations were observed between self-efficacy levels and most dermatological manifestations, including ulceration (χ^2^ = 25.07, *p* < 0.001), swelling (χ^2^ = 10.02, *p* = 0.040), yellow nails (χ^2^ = 29.74, *p* < 0.001), slow nail growth (χ^2^ = 29.44, *p* < 0.001), itching (χ^2^ = 47.01, *p* < 0.001), insomnia (χ^2^ = 61.60, *p* < 0.001), tenderness (χ^2^ = 23.77, *p* < 0.001), redness (χ^2^ = 19.08, *p* = 0.001), paronychia (χ^2^ = 35.66, *p* < 0.001), skin hyperpigmentation (χ^2^ = 23.78, *p* = 0.001), pus formation (χ^2^ = 19.55, *p* = 0.001), and rashes (χ^2^ = 50.75, *p* = 0.001). In contrast, vascular impairment showed no statistically significant association with self-efficacy levels (χ^2^ = 5.39, *p* = 0.249). Overall, patients with higher self-efficacy tended to demonstrate different distributions of dermatological manifestations compared with those reporting low or moderate self-efficacy, highlighting a significant relationship between self-efficacy and most diabetes-related skin conditions.

**Table 6:** Association between self-efficacy levels and dermatological manifestations among patients with type 2 diabetes mellitus (*n* = 2,011).

Skin disorder manifestations	Low (<50%) (*n* = 465)		Moderate (50–75%) (*n* = 617)		High (>75%) (*n* = 929)		χ^2^	*p*
	*n*	%	*n*	%	*n*	%		
Ulcer								
Always	157	33.8	163	26.4	347	37.4	25.07	< 0.001^*^
Sometimes	145	31.2	179	29.0	257	27.7		
Never	163	35.1	275	44.6	325	35.0		
Swelling								
Always	152	32.7	183	29.7	317	34.1	10.02	0.040^*^
Sometimes	145	31.2	231	37.4	279	30.0		
Never	168	36.1	203	32.9	333	35.8		
Yellow nails								
Always	183	39.4	199	32.3	262	28.2	29.74	< 0.001^*^
Sometimes	164	35.3	188	30.5	352	37.9		
Never	118	25.4	230	37.3	315	33.9		
Slow nail growth								
Always	165	35.5	186	30.1	351	37.8	29.44	< 0.001^*^
Sometimes	134	28.8	254	41.2	272	29.3		
Never	166	35.7	177	28.7	306	32.9		
Itching								
Always	138	29.7	200	32.4	389	41.9	47.01	< 0.001^*^
Sometimes	211	45.4	246	39.9	265	28.5		
Never	116	24.9	171	27.7	275	29.6		
Insomnia								
Always	101	21.7	255	41.3	319	34.3	61.60	< 0.001^*^
Sometimes	194	41.7	204	33.1	269	29.0		
Never	170	36.6	158	25.6	341	36.7		
Tenderness								
Always	165	35.5	221	35.8	416	44.8	23.77	< 0.001^*^
Sometimes	123	26.5	199	32.3	244	26.3		
Never	177	38.1	197	31.9	269	29.0		
Redness								
Always	189	40.6	228	36.9	320	34.4	19.08	0.001^*^
Sometimes	117	25.2	223	36.1	308	33.2		
Never	159	34.2	166	26.9	301	32.4		
Paronychia								
Always	137	29.5	240	38.9	246	26.5	35.66	< 0.001^*^
Sometimes	166	35.7	209	33.9	409	44.0		
Never	162	34.8	168	27.2	274	29.5		
Vascular impairment								
Always	163	35.1	237	38.4	368	39.6	5.39	0.249
Sometimes	140	30.1	156	25.3	257	27.7		
Never	162	34.8	224	36.3	304	32.7		
Skin hyperpigmentation								
Always	162	34.8	266	43.1	337	36.3	23.78	0.001^*^
Sometimes	185	39.8	177	28.7	287	30.9		
Never	118	25.4	174	28.2	305	32.8		
Pus								
Always	153	32.9	201	32.6	275	29.6	19.55	0.001^*^
Sometimes	145	31.2	186	30.1	370	39.8		
Never	167	35.9	230	37.3	284	30.6		
Rashes								
Always	182	39.1	156	25.3	216	23.3	50.75	0.001^*^
Sometimes	173	37.2	234	37.9	358	38.5		
Never	110	23.7	227	36.8	355	38.2		

**χ^2^**, Chi square test; ***p*, **Statistically significant at *p* ≤ 0.05. ^*^Means statistically significant.

Table 7 presents the multiple linear regression analysis of factors associated with self-efficacy among patients with Type 2 Diabetes Mellitus. The findings demonstrated that skin care practices and dermatological manifestations were significant positive predictors of self-efficacy. Higher educational level and longer duration of diabetes were also significantly associated with increased self-efficacy levels, whereas increasing age was negatively associated with self-efficacy. Gender showed a significant association, with females reporting higher self-efficacy compared with males. The overall regression model was statistically significant and explained 42% of the variance in self-efficacy scores.

**Table 7:** Multiple linear regression analysis of factors associated with self-efficacy among patients with type 2 diabetes mellitus (*n* = 2,011).

Independent variables	B	SE	β	*t*	*p*-value	95% CI
Age	−0.214	0.062	^−^0.118	^−^3.45	0.001^*^	^−^0.336 to −0.092
Gender	1.842	0.541	0.109	3.40	0.001^*^	0.781 to 2.903
Educational level	2.315	0.488	0.176	4.74	< 0.001^*^	1.358 to 3.272
Duration of diabetes	1.126	0.433	0.092	2.60	0.009^*^	0.278 to 1.974
Dermatological manifestations score	0.845	0.137	0.221	6.17	< 0.001^*^	0.576 to 1.114
Skin care practices score	1.964	0.251	0.308	7.82	< 0.001^*^	1.472 to 2.456

B, Unstandardized regression coefficient; SE, Standard Error; β, Standardized beta coefficient; CI, Confidence Interva; R^2^, Coefficient of determination. ^*^Statistically significant at *p* < 0.05.

## 4 Discussion

Concerning sociodemographic characteristics, the present study demonstrated a slight predominance of males over females, with most participants being Saudi nationals, reflecting the demographic profile of the study setting. Most participants were employed, while a relatively limited proportion had attained university-level education. These findings are generally consistent with previous studies conducted among patients with Type 2 Diabetes Mellitus in similar populations. However, the relatively lower proportion of participants with higher educational attainment contrasts with findings reported by Altubouli and Ali (11) and Mohialdeen et al. (10), who observed higher educational levels associated with improved diabetes self-management and health literacy (10, 11). Furthermore, Kack et al. (12) and Edwards and Yosipovitch (13) reported inconsistent associations between educational level and diabetes-related outcomes, suggesting that structured educational programs may reduce disparities related to educational background (12, 13). Lower educational attainment may negatively affect patients' awareness, adherence to treatment recommendations, and self-management behaviors.

Regarding clinical characteristics, a considerable proportion of participants had a diabetes duration ranging from 7–10 years, with most receiving insulin therapy. This finding may reflect disease progression and the need for intensified glycemic management over time. Similar findings were reported by Gupta et al. (14) and Lima et al. (15), who associated longer disease duration with increased insulin dependence due to declining β-cell function (14, 15). Concerning glycemic control, many participants demonstrated suboptimal HbA1c and blood glucose levels, indicating inadequate metabolic control. These findings are consistent with Kuang et al. (16), who reported that a substantial proportion of patients with T2DM fail to achieve recommended glycemic targets (16). In contrast, Masoompour et al. (17) and Naguib et al. (3) reported better glycemic outcomes in settings where structured diabetes education and comprehensive disease-management programs were implemented (17, 18). These differences may be attributed to variations in healthcare accessibility, patient education, adherence to treatment regimens, and continuity of follow-up care.

Regarding dermatological manifestations, the present study revealed a high prevalence and wide variability of skin-related conditions among patients with Type 2 Diabetes Mellitus. Symptoms such as itching, tenderness, redness, nail abnormalities, ulceration, and paronychia were commonly reported, reflecting the substantial burden of diabetes-related skin complications. The high frequency of these manifestations may be attributed to poor glycemic control, impaired immune response, peripheral neuropathy, and microvascular dysfunction commonly associated with diabetes. Similar findings were reported by Ly et al. (19) and Văţă et al. (19), who documented a high prevalence of dermatological manifestations among patients with diabetes (19, 20).

The observed variability in symptom frequency may reflect differences in disease severity, glycemic status, duration of diabetes, and adherence to self-care practices among participants. Additionally, manifestations such as ulceration and vascular impairment may indicate the progression of underlying neuropathic and microvascular complications associated with long-standing diabetes. In contrast, Qiu et al. (20) reported lower prevalence rates of certain dermatological conditions, which may be explained by variations in population characteristics, diagnostic methods, healthcare accessibility, and disease management approaches (21). Differences in assessment methods, particularly the use of self-reported symptoms vs. clinical dermatological examination, may also contribute to discrepancies across studies. Overall, the findings emphasize the multifactorial nature of diabetes-related skin complications and highlight the importance of early dermatological assessment, glycemic control, and comprehensive preventive care among patients with T2DM.

Regarding skin care practices, the present study demonstrated relatively favorable adherence to certain routine preventive behaviors, particularly regular foot examination, daily washing, and personal hygiene practices. These findings may reflect an acceptable level of awareness regarding diabetic skin and foot care among participants. Similar findings were reported by Naguib et al. (3), who observed relatively good adherence to basic preventive skin care behaviors among patients with Type 2 Diabetes Mellitus, particularly in populations with access to health education programs (3, 18). Nevertheless, important gaps remained in several protective behaviors. The low proportion of participants who reported regular sock use and the inconsistent use of disinfectants before insulin injection may increase the risk of skin injury, infection, and diabetic foot complications.

These findings are consistent with Kalra et al. (21), who reported suboptimal adherence to comprehensive diabetic foot care despite acceptable awareness of basic preventive measures (22). In contrast, Adeyemi et al. (22) demonstrated higher adherence to preventive self-care behaviors in settings implementing structured educational interventions and continuous patient follow-up (23). The variability observed in skin care practices may be influenced by differences in health literacy, patient education, access to healthcare services, and continuity of diabetes counseling programs. Although the current findings indicate acceptable adherence to some aspects of skin and foot care, the persistence of gaps in protective and preventive practices highlights the need for targeted educational interventions aimed at improving comprehensive self-care behaviors and reducing diabetes-related dermatological complications.

Regarding the association between sociodemographic characteristics and self-efficacy, the present study demonstrated significant relationships between self-efficacy and age, gender, educational level, and duration of diabetes among patients with Type 2 Diabetes Mellitus, whereas occupation showed no significant association. Younger participants demonstrated higher self-efficacy levels, which may be attributed to greater adaptability, increased engagement in self-care behaviors, and easier access to health-related information and digital educational resources. Similar findings have been reported in previous studies; however, Sharoni et al. (23) and Tokmakova et al. (24) reported no significant relationship between age and self-efficacy, suggesting that accumulated disease-management experience among older individuals may partially compensate for age-related limitations (24, 25).

Gender differences were also observed, with females demonstrating higher self-efficacy levels than males. This finding is consistent with Wendling and Beadle (25), who suggested that women are generally more likely to engage in health-promoting behaviors and adhere to treatment recommendations (26). In contrast, Fereidooni et al. (26) reported either no significant gender differences or higher self-efficacy among males, which may reflect sociocultural differences and variations in healthcare-seeking behaviors across populations (27). Educational level showed a strong positive association with self-efficacy, supporting previous evidence that higher educational attainment associated with health literacy, problem-solving abilities, and chronic disease-management skills.

Similar findings were reported by Mohialdeen et al. (10), who emphasized the role of education in enhancing diabetes self-management and treatment adherence (10). However, Masoompour et al. (17) reported weaker associations in settings where structured diabetes education programs were broadly accessible regardless of educational background, indicating that continuous patient education may reduce disparities related to formal educational attainment (17). In contrast, occupation was not significantly associated with self-efficacy, suggesting that employment status alone may not directly influence patients' confidence in managing diabetes-related self-care activities. Similar findings were reported by Sharoni et al. (23), who identified inconsistent associations between occupational status and self-efficacy (24). Furthermore, the positive association between longer diabetes duration and higher self-efficacy observed in the current study may reflect accumulated disease-management experience, adaptation, and increased familiarity with treatment regimens over time. Nevertheless, Fereidooni et al. (26) reported lower self-efficacy among patients with prolonged disease duration due to treatment fatigue, complications, and psychological burden, highlighting the complex and multifactorial nature of this relationship (27).

Regarding the association between self-efficacy and dermatological manifestations, the present study demonstrated significant relationships between self-efficacy levels and most skin-related conditions among patients with Type 2 Diabetes Mellitus. Manifestations such as ulcers, itching, insomnia, paronychia, nail abnormalities, and rashes were significantly associated with self-efficacy levels. These findings may suggest that patients experiencing visible or symptomatic complications become more engaged in self-management behaviors and preventive care practices, thereby improving their confidence in managing their condition. Similar findings were reported by Chen and Wu (27), who suggested that the presence of diabetes-related complications may increase patients' motivation to adopt self-care behaviors and strengthen self-management confidence.

In contrast, Dehghan et al. (9) reported lower self-efficacy among patients experiencing chronic complications, suggesting that complications may contribute to psychological distress, functional limitations, and reduced confidence in disease management (9). These discrepancies may be related to differences in disease severity, healthcare accessibility, patient education, psychosocial adaptation, and availability of diabetes support programs across study populations. Additionally, the observed associations between self-efficacy and manifestations such as itching, skin infections, and nail abnormalities may be explained by the influence of poor glycemic control, peripheral neuropathy, and microvascular dysfunction on both skin integrity and patients' daily self-care behaviors. Patients experiencing recurrent or distressing symptoms may become more attentive to preventive practices and disease management activities. Interestingly, vascular impairment was not significantly associated with self-efficacy levels, suggesting that less visible or less immediately symptomatic complications may exert weaker influence on patients' perceived ability to manage their disease compared with more distressing dermatological manifestations.

Regarding predictors of self-efficacy, the multiple linear regression analysis demonstrated that skin care practices and dermatological manifestations were significant predictors of self-efficacy among patients with T2DM. Participants who reported better skin care practices exhibited higher self-efficacy levels, highlighting the important role of preventive self-care behaviors in strengthening patients' confidence in disease management. Similar findings were reported by Chen and Wu (27) and Mohialdeen et al. (10), who emphasized that adherence to diabetes self-care practices and improved health literacy significantly enhance self-efficacy and chronic disease management outcomes (12, 28). Furthermore, the positive association between dermatological manifestations and self-efficacy observed in the current study may suggest that patients experiencing visible or symptomatic complications become more attentive to disease management and preventive care behaviors. Educational level and longer duration of diabetes also demonstrated positive associations with self-efficacy/ Similar findings were reported by Wendling and Beadle (25), who highlighted the role of education and self-management experience in improving self-efficacy among patients with chronic diseases (26).

In contrast, Dehghan et al. (9) and Fereidooni et al. (26) reported lower self-efficacy among patients experiencing diabetes-related complications and prolonged disease duration, suggesting that chronic complications may contribute to psychological distress, treatment fatigue, and reduced confidence in self-management (9, 27). These discrepancies may be explained by differences in disease severity, psychosocial support, healthcare accessibility, educational interventions, and cultural factors across study populations. Overall, the current findings reinforce the importance of structured diabetes education, continuous self-management support, and early dermatological assessment in improving self-efficacy and reducing diabetes-related complications.

### 4.1 Study limitations

Despite the strengths of this study, several limitations should be acknowledged. First, the cross-sectional design limits the ability to establish causal relationships between self-efficacy and dermatological manifestations; therefore, the findings should be interpreted as associative rather than causal. Second, using a self-reported online questionnaire, which may be subject to recall bias and social desirability bias, potentially affecting the accuracy of responses. Third, the use of convenience sampling may limit the generalizability of the findings, despite the relatively large sample size and broad geographic coverage. Additionally, dermatological manifestations were assessed using self-reported data rather than objective clinical examination, which may have introduced reporting bias and limited diagnostic accuracy. Finally, potential confounding variables such as comorbidities, treatment adherence, and psychological factors were not fully explored.

## 5 Conclusion

This study demonstrated a significant association between self-efficacy and dermatological manifestations among patients with T2DM. A high prevalence of skin-related conditions was observed, highlighting the substantial burden of dermatological complications in this population. Sociodemographic factors, particularly age, gender, educational level, and duration of diabetes, were significantly associated with self-efficacy, while occupation showed no significant relationship. Additionally, inconsistencies in adherence to preventive skin care practices were identified. These findings underscore the critical role of self-efficacy in diabetes self-management and its potential influence on the occurrence and management of dermatological complications.

### 5.1 Implications for practice and research

The findings of this study have important implications for clinical practice, nursing education, and public health interventions.

- First, healthcare providers should prioritize patient-centered educational programs that enhance self-efficacy and promote comprehensive skin care practices among patients with diabetes.
- Second, targeted interventions should be developed to address gaps in preventive behaviors, particularly those related to infection control and protective care.
- Third, integrating dermatological assessment into routine diabetes care may facilitate early detection and management of complications, thereby reducing morbidity.
- From a research perspective, future studies should explore the causal pathways between self-efficacy and dermatological outcomes using longitudinal or interventional designs.
- Additionally, incorporating objective clinical assessments and evaluating the impact of structured educational programs on self-efficacy and clinical outcomes would further strengthen the evidence base.
- Finally, multidisciplinary approaches involving nursing, dermatology, and public health are recommended to optimize patient outcomes and quality of life among individuals with diabetes.

## Data Availability

The original contributions presented in the study are included in the article/supplementary material, further inquiries can be directed to the corresponding author.
